# Neutrophil counts distinguish between malignancy and arthritis in children with musculoskeletal pain: a case–control study

**DOI:** 10.1186/1471-2431-13-15

**Published:** 2013-01-31

**Authors:** Antonella Agodi, Martina Barchitta, Cristina Trigilia, Patrizia Barone, Silvia Marino, Rosaria Garozzo, Manuela La Rosa, Giovanna Russo, Andrea Di Cataldo

**Affiliations:** 1 Department “GF Ingrassia”, University of Catania, Via Santa Sofia 87, Catania, 95123, Italy; 2 Unit of Paediatric Haematology and Oncology, Department of Paediatrics, University of Catania, Via Santa Sofia 78, Catania, 95123, Italy; 3 Unit of Paediatric Day Hospital, Department of Paediatrics, University of Catania, Via Santa Sofia 78, Catania, 95123, Italy

**Keywords:** ALL neuroblastoma, Juvenile idiopathic arthritis, Pediatric hematology/oncology, Musculoskeletal pain, White blood cell count

## Abstract

**Background:**

To identify the predictive factors for malignancies using basic clinical and laboratory information in children presenting with musculoskeletal pain and eventually diagnosed with juvenile idiopathic arthritis (JIA) or malignancy.

**Methods:**

A retrospective case–control chart review research examining laboratory data from patients referred for musculoskeletal pain in 2001–2010 and diagnosed with malignancy or JIA was performed. The validity of each test for the diagnosis of neoplasia was assessed by calculating the sensitivity, specificity, positive predictive values (PPV), negative predictive values (NPV) and likelihood ratios.

**Results:**

A total of 134 patients were enrolled. Statistically significant differences were found in neutrophil count, Hb, LDH, IgA and C4 values, ANA, anti-EA EBV IgG and anti-CMV IgG titres. High LDH value and anti-CMV IgG were the most predictive factors for neoplasia. High specificity factors for neoplasia were abnormal values of neutrophil count, Hb, IgA and C4, and the presence of anti-EA EBV and anti-CMV IgG. High PPV were recorded for abnormal neutrophil count, Hb value and anti-CMV titre. A low NPV was found only for anti-EA EBV and anti-CMV titres.

**Conclusions:**

In this setting of patients, minimum changes in neutrophil count, particularly if associated with low Hb and high LDH levels, are to be thoroughly considered, because they appear as the most predictive factors for the diagnosis of tumour.

## Background

Musculoskeletal pain is a common symptom in children, and in some cases it is the first sign of a severe disease such as chronic inflammatory disease or a malignancy [1-4]. About 15% to 30% of children with acute lymphoblastic leukaemia (ALL), the commonest childhood malignancy, and most patients with disseminated neuroblastoma report joint and/or bone symptoms [2-4]. In a child with leukaemia, musculoskeletal pain is normally accompanied by other clinical, laboratory and imaging data that easily indicate the diagnosis. Only in very few cases this sign is isolated, which complicates the diagnosis [2,4]. On the other hand, in the absence of patognomonic tests the diagnosis of juvenile idiopathic arthritis (JIA) is the result of an integrative analysis of several data excluding other potential aetiologies of the pain and satisfying the established criteria [5]. Adult patients are able to guide the physician to the right diagnosis since they can thoroughly depict the characteristics of the pain. Conversely, a child can hardly describe it clearly.

In this particular setting, the insidious onset and the lack of typical signs and laboratory tests may delay malignancy diagnosis in children [5,6]. Another risk is misdiagnosis, like JIA, and the consequent administration of corticosteroids leading to further delays and wrong treatments [6].

Literature data comparing children with malignancies to those with JIA for the pattern of initial findings is limited and involves small series of patients [7-9] and to translate results into optimal clinical practice is crucial: this is the reason why we undertook this retrospective analysis.

In order to improve the quality of clinical practice and patient outcomes, the aim of our study was to identify the predictive factors for malignancies using basic clinical and laboratory information in children presenting with musculoskeletal pain and eventually diagnosed with JIA or malignancy.

## Methods

The study is a retrospective case–control chart review research examining both clinical and laboratory data from the initial visit of all the patients referred for musculoskeletal pain to the Department of Paediatrics of the University of Catania during the period January 2001–December 2010 and diagnosed with a neoplastic disease or JIA. We included patients with neoplasia and patients with JIA, already diagnosed, and retrospectively reviewed their charts in order to evaluate all relevant clinical and laboratory data. Patients with blast cells in the blood smear were excluded.

The study has been approved by the Institutional Review Board of our Department of Pediatrics.

Information regarding demographic data, symptoms, clinical manifestations, diagnosis, outcome and laboratory data were collected from the clinical records.

Statistical analyses were performed using the software SPSS version 14.0 (SPSS, Chicago, IL, USA). Percentage was used to describe categorical variables, while continuous variables were described by mean and range. Statistical comparison of proportions was performed by *χ*^2^ test; statistical comparison of continuous variables was carried out by Student’s *t*-test. Basic significance level was fixed at p <0.05. The value of the given diagnostic test as a predictive factor for neoplasia in comparison to JIA and for ALL in comparison to JIA were assessed among groups, only for significantly different variables (*p* <0.05), by calculating sensitivity, specificity, positive predictive value (PPV), negative predictive value (NPV), positive and negative likelihood ratios (LR+ and LR- respectively). In particular, for each diagnostic test, sensitivity was determined by calculating the proportion of the number of true positive patients among all patients with the disease (i.e. true positives and false negatives); specificity was determined by calculating the proportion of the number of true negative patients among all healthy patients (i.e. true negatives and false positives); PPV was determined by calculating the proportion of the number of true positive patients among all positive patients (i.e. true positives and false positives); NPV was determined by calculating the proportion of the number of true negative patients among all negative patients (i.e. true negatives and false negatives); the LR+ was computed as the sensitivity divided by 1–specificity and the LR- as 1–sensitivity divided by specificity. The differences in the denominators were the result of missing data for the variable analyzed.

## Results

During the ten-year study a total of 150 patients hospitalised because of musculoskeletal pain were eventually diagnosed with JIA (*n* = 71) or malignancy (*n* = 79). Sixteen out of the 79 children with malignancy were excluded because blast cells were shown in the blood smear, so 134 patients (63 with malignancy and 71 with JIA) were finally enrolled in the study. Their main characteristics are shown in Table 1. ALL was the most frequent neoplasia type and oligoarticular JIA the most frequent JIA subtype. Age and gender distributions were similar in the two groups.

**Table 1:** Characteristics of patients with neoplasia and patients with JIA

	**All patients (n= 134)**	**Neoplasia (n= 63)**	**JIA (n= 71)**
Mean age in months (range)	92.2 (3–203)	92.6 (3–203)	91.9 (15–198)
Female	57.5%	52.4%	62.0%
Neoplasia types			
- ALL		47 74.6%	
- Lymphomas		4 6.3%	
- Other neoplasia		12 19.1%	
JIA subtypes			
- systemic			18 25.4%
- polyarthritis			15 21.1%
- oligoarticular			36 50.7%
- psoriasis			1 1.4%
- other			1 1.4%

Normal reference laboratory values, relative to the limits of the norm of our Institution, are indicated in Table 2 together with the number and percentage of patients with low and high values for each laboratory test, both for the neoplasia and the JIA groups. Furthermore, the comparison of laboratory tests as diagnostic markers of patients in the neoplasia group and in the JIA group, considered as absolute number and percentage of patients with abnormal values, is reported in Table 3. Statistically significant differences in the neoplasia and the JIA groups were found between the following parameters: neutrophils count, haemoglobin (Hb), lactate dehydrogenase (LDH), IgA and C4 subunit of complement (C4) values, antinuclear antibodies (ANA), anti-early antigen (EA) Epstein-Barr virus (EBV) IgG and anti-cytomegalovirus (CMV) IgG titres. No significant differences were found in the other parameters under evaluation and for calcium, phosphorus, anti-muscle antibodies (AMA) and anti-smooth muscle antibodies (ASMA). High LDH value and positivity to anti-CMV IgG were the most predictive factors for neoplasia (sensitivity >70%). High specificity (≥ 80%) for neoplasia was shown by the following factors: abnormal values of neutrophil count, Hb, IgA and C4, and the presence of anti-EA EBV and anti-CMV IgG. High PPVs (≥ 85%) were recorded for abnormal neutrophil count, Hb value and anti-CMV IgG titre. A low NPV (< 50%) was found only for anti-EA EBV and anti-CMV IgG titres. Neutrophils count, Hb, IgA, C4 and anti-CMV IgG titre, shown high LR+ values (>2).

**Table 2:** Normal values of diagnostic markers in study

**Diagnostic markers**	**Normal value (reference value)**	**Neoplasia (N=63)**			**JIA (N=71)**		
		**Lower values or presence (n/N; %)**	**Normal values (n/N; %)**	**Higher values or positivity (n/N; %)**	**Lower values or present (n/N; %)**	**Normal values (n/N; %)**	**Higher values or positivity (n/N; %)**
WBC count	4-10 × 10^9^/L	11/60; 18.3%	27/60; 45%	22/60; 36.7%	3/68; 4.4%	35/68; 51.5%	30/68; 44.1%
Neutrophil count	1 × 10^9^/L	25/55; 45.5%	30/55; 54.5%	0/55; 0%	2/61; 3.3%	59/61; 96.7%	0/61; 0%
Platelet count	150-300 × 10^9^/L	34/60; 56.7%	13/60; 21.7%	13/60; 21.7%	2/68; 2.9%	17/68; 25%	49/68; 72.1%
Hemoglobin (Hgb)	≥10 gr/dl	34/60; 56.7%	26/60; 43.3%	NA	62/68; 91.2%	6/68; 8.8%	NA
Lactic dehydrogenase (LDH)	<500 IU/L	NA	7/58; 12.1%	51/58; 87.9%	NA	16/49; 32.7%	33/49; 67.3%
Erythrocyte sedimentation rate (ESR)	<20 mm/h	NA	4/37; 10.8%	33/37; 89.2%	NA	14/68; 20.6%	54/68; 79.4%
C-reactive protein (CRP)	<1 mg/dl	NA	13/52; 25%	39/52; 75%	NA	14/61; 23%	47/61; 77%
Antinuclear antibody (ANA)	≥1:80	NA	16/19; 84.2%	3/19; 15.8%	NA	30/62; 48.4%	32/62; 51.6%
IgG	751–1560 mg/dl	9/51; 17.6%	35/51; 68.6%	7/51; 13.7%	2/57; 3.5%	35/57; 61.4%	20/57; 35.1%
IgM	46–304 mg/dl	4/51; 7.8%	47/51; 92.2%	0/51; 0%	1/57; 1.8%	53/57; 93%	3/57; 5.3%
IgA	82–453 mg/dl	17/51; 33.3%	33/51; 64.7%	1/51; 2%	6/57; 10.5%	49/57; 86%	2/57; 3.5%
C3	79–152 mg/dl	1/36; 2.8%	14/36; 38.9%	21/36; 58.3%	3/54; 5.6%	29/54; 53.7%	22/54; 40.7%
C4	16–38 mg/dl	1/36; 2.8%	19/36; 52.8%	16/36; 44.4%	4/53; 7.5%	44/53; 83%	5/53; 9.4%
Anti-EBV IgM	Negative	NA	37/44; 84.1%	7/44; 15.9%	NA	13/14; 92.9%	1/14; 7.1%
Anti-EBV IgG	Negative	NA	11/44; 25%	33/44; 75%	NA	7/15; 46.7%	8/15; 53.3%
Anti-EA EBV IgG	Negative	NA	21/35; 60%	14/35; 40%	NA	10/10; 100%	0/10; 0%
Anti-CMV IgM	Negative	NA	32/34; 94.1%	2/34; 5.9%	NA	8/10; 80%	2/10; 20%
Anti-CMV IgG	Negative	NA	9/33; 27.3%	24/33; 72.7%	NA	8/10; 80%	2/10; 20%

**Table 3:** Comparison of abnormal values of diagnostic markers between neoplasia and JIA patients

**Diagnostic markers**	**All patients n/N (%)**	**Neoplasia n/N (%)**	**JIA n/N (%)**	***p*-value (Neoplasia versus JIA)**	**Sensitivity**	**Specificity**	**PPV**	**NPV**	**LR+**	**LR-**
WBC count	66/128 (51.6)	33/60 (55.0)	33/68 (48.5)	0.484						
Neutrophil count	27/116 (23.3)	25/55 (45.5)	2/61 (3.3)	0.000	45.5%	96.7%	92.5%	66.2%	13.8	0.6
Platelet count	98/128 (76.6)	47/60 (78.3)	51/68 (75.0)	0.682						
Hb	40/128 (31.3)	34/60 (56.7)	6/68 (8.8)	0.000	56.7%	91.2%	85.0%	70.4%	6.4	0.5
LDH	84/107 (78.5)	51/58 (87.9)	33/49 (67.3)	0.017	87.9%	32.7%	60.7%	69.5%	1.3	0.4
ESR	87/105 (82.9)	33/37 (89.2)	54/68 (79.4)	0.281						
CRP	86/113 (76.1)	39/52 (75.0)	47/61 (77.0)	0.828						
ANA	35/81 (43.2)	3/19 (15.8)	32/62 (51.6)	0.008	15.8%	48.4%	8.5%	65.2%	0.3	1.7
IgG	38/108 (35.2)	16/51 (31.4)	22/57 (38.6)	0.545						
IgM	8/108 (7.4)	4/51 (7.8)	4/57 (7.0)	1.000						
IgA	26/108 (24.1)	18/51 (35.3)	8/57 (14.0)	0.013	35.3%	86.0%	69.2%	59.7%	2.5	0.8
C3	47/90 (52.2)	22/36 (61.1)	25/54 (46.3)	0.200						
C4	26/89 (29.2)	17/36 (47.2)	9/53 (17.0)	0.004	47.2%	83.0%	65.3%	69.8%	2.8	0.6
Anti-EBV IgM	8/58 (13.8)	7/44 (15.9)	1/14 (7.1)	0.665						
Anti-EBV IgG	41/59 (69.5)	33/44 (75.0)	8/15 (53.3)	0.192						
Anti-EA EBV IgG	14/45 (31.1)	14/35 (40.0)	0/10 (0)	0.019	40.0%	100%	1.0%	32.2%	-	0.6
Anti-CMV IgM	4/44 (9.1)	2/34 (5.9)	2/10 (20.0)	0.218						
Anti-CMV IgG	26.43 (60.5)	24/33 (72.7)	2/10 (20.0)	0.007	72.7%	80.0%	92.3%	47.0%	3.6	0.3

The combination of all three abnormal values in neutrophil count, Hb and LDH provide an increase in sensitivity (93.3%) and in specificity (100%).

Table 4 reports the results of diagnostic markers, considered as number and percentage of patients with abnormal values, among patients with ALL compared with those of patients with JIA. Statistically significant differences were found between the two groups with regard to neutrophil count, Hb, IgA and C4 values, ANA, anti-EA EBV and anti-CMV IgG titres. No significant differences were found in the abnormal values of the other parameters under evaluation and for calcium, phosphorus, AMA and ASMA. The most predictive factors for ALL were as follows: with high sensitivity (>70%) positivity to anti-CMV IgG only; with high specificity (≥80%), neutrophil count, Hb value, IgA, C4, anti-EA IgG and anti-CMV IgG titres. High PPVs (>80%) were recorded for neutrophil count, Hb value and anti-CMV IgG titre. NPVs were low (<50%) for anti-EA IgG titre. Neutrophils count, Hb, IgA, C4 and anti-CMV IgG titre, shown high LR+ values (>2).

**Table 4:** Comparison of abnormal values of diagnostic markers between ALL and JIA patients

**Diagnostic markers**	**All patients n/N (%)**	**ALL n/N (%)**	**JIA n/N (%)**	***p*-value (ALL versus JIA)**	**Sensitivity**	**Specificity**	**PPV**	**NPV**	**LR+**	**LR-**
WBC count	59/113 (52.2)	26/45 (57.8)	33/68 (48.5)	0.345						
Neutrophil count	23/101 (22.8)	21/40 (52.5)	2/61 (3.3)	0.000	52.5%	96.7%	91.3%	75.6%	15.9	0.5
Platelet count	87/113 (77.0)	36/45 (80.0)	51/68 (75)	0.650						
Hb	32/113 (28.3)	26/45 (57.8)	6/68 (8.8)	0.000	57.8%	91.2%	81.2%	76.5%	6.6	0.5
LDH	70/92 (76.1)	37/43 (86.0)	33/49 (67.3)	0.050						
ESR	78/95 (82.1)	24/27 (88.9)	54/68 (79.4)	0.379						
CRP	73/99 (73.7)	26/38 (68.4)	47/61 (77.0)	0.358						
ANA	34/76 (44.7)	2/14 (14.3)	32/62 (51.6)	0.016	14.3%	48.4%	5.8%	71.4%	0.3	1.8
IgG	34/96 (35.4)	12/39 (30.8)	22/57 (38.6)	0.517						
IgM	6/96 (6.3)	2/39 (5.1)	4/5 (7.0)	1.000						
IgA	23/96 (24.0)	15/39 (38.5)	8/57 (14.0)	0.008	38.5%	86%	65.2%	67.1%	2.8	0.7
C3	42/82 (51.2)	17/28 (60.7)	25/54 (46.3)	0.250						
C4	23/81 (18.4)	14/28 (50.0)	9/53 (17.0)	0.004	50%	83%	60.8%	75.8%	2.9	0.6
Anti-EBV IgM	8/50 (16.0)	7/36 (19.4)	1/14 (7.1)	0.414						
Anti-EBV IgG	35/51 (68.6)	27/36 (75.0)	8/15 (53.3)	0.187						
Anti-EA EBV IgG	12/40 (30.0)	12/30 (40.0)	0/10 (0)	0.019	40.0%	100%	1.0%	35.7%	-	0.6
Anti-CMV IgM	4/40 (10.0)	2/30 (6.7)	2/10 (20.0)	0.256						
Anti-CMV IgG	24/39 (61.5)	22/29 (75.9)	2/10 (20.0)	0.003	75.9%	80.0%	91.6%	53.3%	3.8	0.3

Comparing patients with ALL to those with JIA, the combination of both abnormal values of neutrophil count and Hb did not show any increase in sensitivity (46.8%), whereas an increase in specificity was observed (98.5%).

As far as clinical data are concerned, monolateral and bilateral musculoskeletal pain were observed with similar frequency in children with JIA (32.4% and 38.0% respectively) and in children with neoplasia (31.7% and 38.1% respectively). Furthermore, fever was significantly more recurrent in children with neoplasia (71.4%) as compared to children with JIA (33.8%) (p = 0.000). Finally, lymphadenopathy was significantly more frequent in children with neoplasia (23.8%) than in children with JIA (7.0%) (p = 0.007).

## Discussion

Musculoskeletal pain is a frequent complaint among children but only in few cases it implies severe diseases such as malignancies or chronic inflammatory diseases. Only in very few cases musculoskeletal pain is an isolated sign hindering the formulation of the diagnosis. In some of those cases general practitioners or paediatricians address the patient to the rheumatologist or the orthopaedist who tend to consider musculoskeletal symptoms as a sign of a chronic inflammatory disease. Therefore they prescribe analyses in order to validate their clinical hypothesis or start treatment without a clear diagnosis. In such a case, treatment with corticosteroids relieves symptoms thus causing a further delay in the diagnosis. Even when both diagnostic hypotheses are included, it can be hard to distinguish the clinical signs of JIA from malignancies because symptoms at the onset may be the same [3,9]. Moreover, in the absence of pathognomonic test the diagnosis of JIA requires the exclusion of all the other causes of musculoskeletal pain. Furthermore, when no haematological signs are present, even leukaemia or a tumour could be difficult to detect. This clinical overlapping explains the need to always exclude a malignancy in children with musculoskeletal symptoms, especially when the clinical pattern is not characteristic of a specific rheumatic disease and/or in the presence of organomegaly or abnormal laboratory or imaging data.

A limited number of studies have been conducted in order to examine the value of diagnostic markers in the differential diagnosis between JIA and malignancies in children [7-9] and results are often inconsistent. In order to identify early parameters useful for a differential diagnosis, clearly defining the role of laboratory tests in terms of usage within the clinical diagnostic algorithms and the patient population for whom they are intended, in the present study we retrospectively evaluated ten-years data of two different groups of children with musculoskeletal pain, one affected by tumour and the other by JIA. No significant difference was found between the two groups for sex and age at the time of the diagnosis. High C-reactive protein (CRP) and erythrocyte sedimentation rate (ESR) were not relevant parameters, being CRP slightly higher among the JIA group, but with no statistically significant difference. Some authors suggest taking the neutrophil count carefully into consideration in a child complaining musculoskeletal pain [2,9]. Our results confirm a statistically significant difference in the neutrophil count between the two groups, being lower values significantly most frequent among patients with tumour (45.5% *vs* 3.3%) or leukaemia (52.5%), with high specificity, PPV and LR+, but low sensitivity. This means that a normal result of this test is very frequent among “non tumour” patients even though an altered result is not a clear sign of a “tumour/leukaemia” patient.

The same results were obtained for the haemoglobin levels: anaemia (i.e. Hb <10 g/dl) was more frequently found in the tumour group (56.7% *vs* 8.8%) or in the leukaemia group (57.8%) with a statistically significant difference. However, it has been reported that anaemia from chronic illness does occur in children with JIA and, for this parameter, we found low sensitivity (56.7%) for tumours. An abnormal platelet count was observed in both groups of patients, with lower values among the tumour group and higher values among the JIA group (p <0.05).

According to literature, high LDH level proved a useful predictive factor for tumours: abnormal values were more prevalent among the tumour group (87.9% vs 67.3%; sensitivity 88.0%). In fact, as previously reported in ALL patients [9], a twofold or even higher increase was found exclusively among children with malignancies. Consequently, if a child complains musculoskeletal pain, high LDH level should be considered suspicious and it requires further analyses to exclude a malignancy.

Moreover, in the comparison between the patients with tumour and those with JIA, the combination of all three abnormal values in neutrophil count, Hb and LDH increased sensitivity and specificity.

We also investigated clinical parameters like fever and lymphadenopathy which resulted significantly more frequent in patients with tumour despite a high number of missing data. However, previous papers have already reported the limited value of these clinical parameters for the comparison of ALL and JIA [9-11].

Some diagnostic tests such as ANA and complement have been reported as not meaningful to differentiate tumours and JIA [9]. In our study the frequency of positive ANA patients was higher among the JIA group and the difference was statistically significant, but this test was characterized by low sensitivity and low specificity. No significant difference in the other screened auto-antibodies appeared between the two groups. Furthermore, no significant difference was detected in the immunoglobulin profile even though we found abnormal IgA values more frequently among the tumour group and this result was statistically significant with high specificity.

An odd result, seemingly difficult to comment, concerns a positive anti-CMV IgG titre found more frequently among patients with tumours than among JIA patients, with a statistically significant difference and with high specificity and high sensitivity, high PPV and low NPV. Our data showed that the presence of these antibodies is the most valid feature in patients with tumour or leukaemia versus patients with JIA, although the low number of tests taken in JIA patients makes the numbers less reliable. Indeed, it has been reported that leukemic patients have a higher titre of antibodies against many herpes viruses than the healthy population [12,13].

## Conclusions

In conclusion, our results suggest that even small changes in the neutrophil count are of critical diagnostic value. In cases where such alterations are associated with low Hb levels and high LDH, the probability of a diagnosis of neoplasia is very elevated. Our study highlights the importance of accurate diagnosis for the appropriate and effective management to improve patient outcomes. Further prospective studies considering a more numerous sample would better evaluate the clinical utility of laboratory tests for the early differential diagnosis of leukaemia/tumour or JIA.

## Abbreviations

JIA: Juvenile idiopathic arthritis; ALL: Acute lymphoblastic leukaemia; RF: Rheumatoid factor; WBC: White blood cells; Hb: Haemoglobin; LDH: Lactate dehydrogenase; ESR: Erythrocyte sedimentation rate; CRP: C-reactive protein; ANA: Antinuclear antibodies; AMA: Antimuscle antibodies; ASMA: Anti smooth muscle antibodies; EBV: Epstein-barr virus; EA: Early antigen; CMV: Cytomegalovirus; PPV: Positive predictive value; NPV: Negative predictive value; NA: Not applicable; LR+: Positive likelihood ratio; LR-: Negative likelihood ratio.

## Competing interest

The authors declare that they have no competing interests.

## Authors‘ contributions

AA conceived and designed the study, was responsible for the data analysis and interpretation and drafted the manuscript; MB participated in the design of the study and performed the data analysis and interpretation and drafted the manuscript; CT participated in the design of the study and performed the data analysis and interpretation and drafted the manuscript; PB participated in the design of the study and performed the data analysis and interpretation and drafted the manuscript; SM participated in the design of the study and performed the data analysis and interpretation and drafted the manuscript; RG participated in the design of the study and performed the data analysis and interpretation and drafted the manuscript; MLR participated in the data analysis and interpretation; GR participated in the design of the study and performed the data analysis and interpretation and drafted the manuscript; ADC conceived and designed the study, was responsible for the data analysis and interpretation and drafted the manuscript. All Authors read and approved the final manuscript.

## Pre-publication history

The pre-publication history for this paper can be accessed here:

http://www.biomedcentral.com/1471-2431/13/15/prepub
